# Regulatory B Cells in Systemic Sclerosis Isolated or Concomitant With Hashimoto Thyroiditis

**DOI:** 10.3389/fimmu.2022.921260

**Published:** 2022-07-06

**Authors:** Silvia Capriello, Silvia Martina Ferrari, Ilenia Gatto, Maria Giulia Santaguida, Poupak Fallahi, Alessandro Antonelli, Giorgio Mangino, Giovanna Romeo, Camilla Virili, Marco Centanni

**Affiliations:** ^1^ Department of Medico-surgical Sciences and Biotechnologies, Endocrinology Section, ‘‘Sapienza’’ University of Rome, Latina, Italy; ^2^ Department of Clinical and Experimental Medicine, University of Pisa, Pisa, Italy; ^3^ Endocrine Unit, Azienda Unità Sanitaria Locale (AUSL) Latina, Latina, Italy; ^4^ Department of Translational Research and New Technologies in Medicine and Surgery, University of Pisa, Pisa, Italy; ^5^ Department of Surgical, Medical and Molecular Pathology and Critical Area, University of Pisa, Pisa, Italy; ^6^ Department of Medico-surgical Sciences and Biotechnologies, Immunology Section, ‘‘Sapienza’’ University of Rome, Latina, Italy

**Keywords:** systemic sclerosis, Hashimoto’s thyroiditis, polyautoimmunity, B regulatory cells, Th17

## Abstract

Systemic sclerosis (SSc) is a systemic autoimmune disease in which gastrointestinal disorders represent a complication in up to 90% of patients. SSc may associate with thyroid autoimmune disorders, with Hashimoto’s thyroiditis (HT) being the more prevalent worldwide. Previous studies have examined the behavior of Th17 lymphocytes and Breg cells in patients with HT and concomitant autoimmune organ-specific disorders. These immune phenotypes seem to play a significant role in the pathogenesis of both these autoimmune processes, but their behavior when these two disorders coexist has not been described. We analyzed Th17 and Breg (CD24hiCD38hi) cell subsets in 50 subjects (45F/5M; median age = 49 years): 18 were healthy donors (HD), 20 had isolated HT, and 12 had SSc, seven of whom had both HT and SSc. Breg cells’ function was also evaluated by measuring their IL-10 production when stimulated by specific activators. An increased percentage of Th17 lymphocytes characterized HT patients as compared to both HD and the whole group of SSc patients (p = 0.0018). On the contrary, the percentage of unstimulated Breg cells in SSc patients was higher (p = 0.0260), either associated or not with HT, as compared to both HT patients and HD, which, instead, showed a similar percentage of Breg cells. Following a specific stimulation with CpG, the percentages of Breg cells were increased in the whole sample of SSc patients (p < 0.001) as well as in isolated SSc and in SSc+HT ones as compared to isolated HT. However, qualitative analysis, obtained through the detection of the IL-10-producing phenotype, revealed that the percentage of CpG-stimulated CD24hiCD38hi-IL10+cells was significantly decreased in SSc patients (p < 0.0001) with no difference between isolated SSc and SSc+HT patients. The IL-10-producing phenotype was instead slightly increased in HT patients as compared to HD (4.1% vs. 2.8%). The presence of SSc seems to be characterized by an enrichment of total Breg cells but by a reduced Breg IL-10-producing phenotype, representing functional Bregs. This last finding was entirely due to the presence of SSc independently from the association with HT. This behavior is different from the ones described about the association of HT with organ-specific autoimmune disorders.

## Introduction

Systemic sclerosis (SSc) is an autoimmune disease in which gastrointestinal disorders represent a highly frequent complication in up to 90% of patients, showing a major site at the level of the upper digestive tract (1). Similarly to other autoimmune disorders, its pathogenesis possibly derives from an imbalance between the self-aggressive and self-tolerant lymphocyte patterns (2). In this view, beside the acquired knowledge about regulatory T cells (Treg), regulatory B cells (Breg) gained interest in the pathogenesis of autoimmune diseases (3, 4). Autoimmune diseases appear to follow a similar dysimmune pathogenic root and the association of multiple autoimmune disorders, both systemic and organ-specific, is not unusual (5, 6). Indeed, a recent study by Rojas et al. (5) observed that polyautoimmunity more often occurs based on the presence of circulating autoantibodies without specific symptoms or signs, and this latent polyautoimmunity was found in about 100% of the patients with SSc. In this context, Hashimoto’s thyroiditis (HT) represents the most common autoimmune disorder worldwide, whose clinical association with coexisting autoimmune diseases has also been described (7–11). A significant association of HT with systemic sclerosis (SSc), regardless of the disease activity, has been described (12). In both HT and SSc, the presence of circulating autoantibodies which constitute the basis for the diagnosis and the proof of B lymphocyte activation (8, 13–15) has been reported. However, in SSc, the autoantibodies may play a pathogenic role while in HT they are mostly considered as an inflammatory epiphenomenon (13, 14). In recent years, several studies have paved the way for new insights into the cellular immune response in this association. Particularly, a polarization of Th17 flag cytokines in both hypothyroid HT and SSc patients during the inflammatory phase has been described (15–17), but the behavior of this pathway when SSc is associated with HT is scarcely known (12, 15, 17). On the contrary, the role of B-effector cells and autoantibodies in isolated SSc is well known, even supported by the therapeutic use of different monoclonal drugs directed against this effector pathway (14, 15, 18, 19). However, recent studies have shown that the overall suppression of B lymphocytes results in the loss of some important regulatory functions mainly supported by Breg cells (15, 18). In HT patients, the Breg-cell percentage has been recently described along with their ability to produce IL10 upon proper stimulation (20–22). Two of these studies examined the distribution and behavior of Breg cells in patients with HT and concomitant autoimmune organ-specific disorders (atrophic gastritis, celiac disease, non-segmental vitiligo) (20, 21). A decreased percentage of Breg cells in SSc has been described as well (23). No studies, instead, characterized the behavior of the Th17 pathway and of Breg cells in patients with SSC associated with HT, and this was the aim of the present study.

## Patients and Methods

### Patients

**Figure 1:**
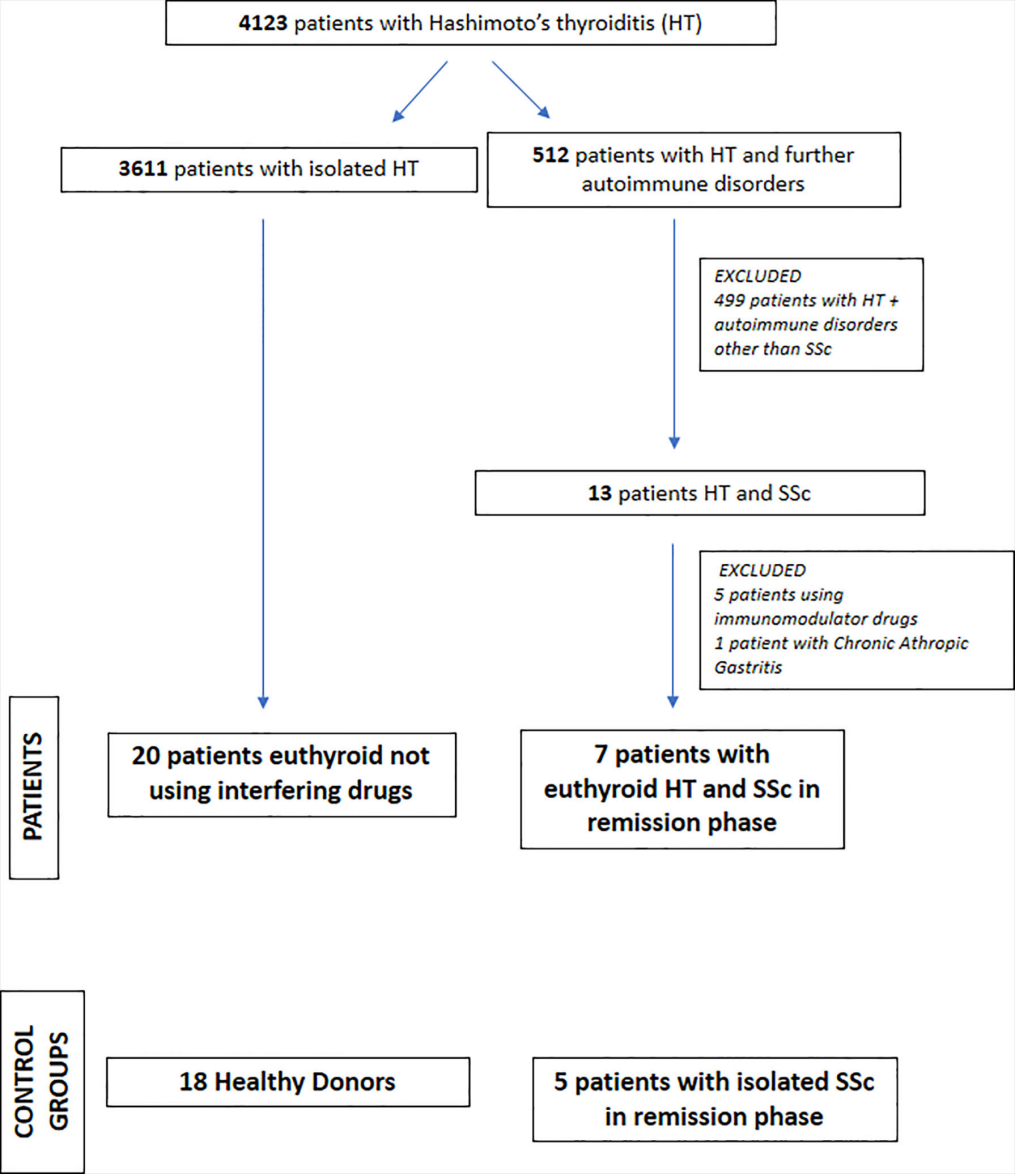
Flow diagram of patients’ selection.

A total of 50 subjects (45 women and 5 men; median age = 49 years) were enrolled: 18 were healthy donors (HD), 20 patients had isolated HT, and 12 patients had SSc, of whom seven also had HT. **Figure 1** depicts the flow diagram of the patient and control group selection; their anthropometric and biochemical characteristics are reported in **Table 1**. The diagnoses were established based on the presence of high titers of anti-thyroid peroxidase antibodies (AntiTPOAb) and pathognomonic ultrasonographic pattern (10). The diagnosis of SSc was defined on the basis of diagnostic criteria described in Denton et al. (1), and confirmed by serologic and histological tests.

**Table 1:** Anthropometric and biochemical data of patients.

	HD n = 18	HTN = 20	SSC n = 12	p
**Age** **(years)**	**46**	**48**	**57**	ns
**Sex** **(F/M)**	**16/2**	**18/2**	**11/1**	
**TSH (mU/l)** **IQR**	**2.1** (1.1-2.5)	**1.3** (0.9-1.9)	**1.5** (0.7-2.2)	ns
**FT_4_ (ng/dL)** **IQR**	**1.1** (1.0-1.3)	**1.3** (1.1-1.6)	**1.2** (1.1-1.4)	ns
**Duration HT** **(years)**	N/A	**8**	N/A	
**Duration SSc (years)**	N/A	N/A	**12**	

The results are expressed as median values (IQR).

At the time of sampling, the median duration of HT patients was 5 years (IQR= 3-9 years) and that of hypothyroidism was 3 years (IQR = 1.8–5 years), and all HT patients were euthyroid. Two euthyroid patients out of 27 were not treated while the remaining 25/27 were treated with an individually tailored dose of levothyroxine (LT4), as previously described (24). In the group with SSc, eight out of 12 showed mild GI manifestations, mostly at the esophagus and small intestine levels. All the seven patients with HT+SSc needed levothyroxine treatment, at an increased LT4 dose as described for other GI disorders (24–27).

In the whole cohort, the following exclusion criteria were considered: i) chronic disorders (diabetes, chronic obstructive pulmonary disease, obesity, cancer, renal failure, HIV/HBV/HCV infections, autoimmune disorders other than HT and SSc); ii) infections and/or inflammatory disorders in the last 6 months; iii) pregnancy or breastfeeding; iv) therapies with drugs that interfere and/or modulate the normal response of the immune system (steroids, anti-inflammatory drugs, immune-suppressant and immune-modulatory, acetylsalicylic acid); v) hormone therapies with estrogen, progesterone, or antiandrogens; vi) recent vaccinations and/or patients taking immune modulators.

All patients were informed about the purpose of the study and gave a written informed consent. The study has been approved by the local Ethical Committee (ASL C, Roma-Latina, Italy) according to the local ethical rules and to the guidelines of the Declaration of Helsinki.

### Methods

All samples were freshly collected by a morning blood withdrawal. Peripheral blood mononuclear cells (PBMCs) were isolated from whole blood by Ficoll-Hypaque (Lympholyte-H) density gradient centrifugation (CL5020, Cedarlane Laboratories Ltd., Burlington, Canada) and used immediately. Effector Th17 was obtained from overnight-stimulated PBMCs with PMA, ionomycin, brefeldin A, and monensin (Affymetrix eBioscience Cell Stimulation Cocktail plus protein transport inhibitor). Subsequently, all cells were stained with anti-CD45 PerCP- and anti-CD4 FITC-conjugated antibodies. Cells were then fixed, permeabilized, and stained with anti-IL-17A PE (Affymetrix eBioscience, San Diego, CA, USA). The Th17 cellular subset was phenotypically identified as CD4^+^IL17A^+^, and its value was expressed as percentage of total CD4^+^ T lymphocytes.

Freshly isolated PBMCs were surface-stained with anti-CD45 PerPC and anti-CD19 FITC to detect total circulating B cells and with anti-CD24 APC-eFluor 780 and anti-CD38 APC to detect the Breg subpopulation (Affymetrix eBioscience, San Diego, CA, USA). Therefore, it was possible to evaluate total CD19^+^ B cells as well as CD19^+^CD24^hi^CD38^hi^ Breg cells.

The different B-cell subpopulations were expressed as percentages of total CD19^+^ B lymphocytes.

To detect IL10 production in B cells, PBMCs were incubated with 0.1 µM CpG type B ODN2006 (*In vivo*Gen, San Diego, CA, USA) for 72 h at 37°C and subsequently stimulated with PMA, ionomycin, brefeldin A, and monensin (Affymetrix eBioscience Cell Stimulation Cocktail plus protein transport inhibitor) in the last 5 h, then collected and stained. PBMCs were similarly treated with non-CpG ODN2006 control (ODN 2137, InvivoGen, San Diego, CA, USA) in order to have a negative control.

PBMCs were surface-stained with anti-CD19FITC, anti-CD24 APC eFluor, and anti-38 APC, fixed, permeabilized, and then stained intracellularly with anti-IL-10 PE (Affymetrix eBioscience, San Diego, CA, USA). Data were expressed as percentages of total IL10^+^ B lymphocyte subpopulations. For gate setting, appropriate isotype control Abs were used for each intracellular staining (Affymetrix eBioscience, San Diego, CA, USA).

Cells were acquired on a FACSAria II flow cytometer (Becton Dickinson, San Jose, CA, USA). At least 10,000 events were acquired on CD4^+^ or CD19^+^ gates to enumerate Th17 and Breg cells, respectively. FACSDiva software (v6.1.1, Becton Dickinson) was used for sample acquisition and analysis.

### Statistical Analysis

Results are expressed as median values. The difference between two groups was calculated using the non-parametric Mann–Whitney U test or paired T test where appropriate. When comparing more than two groups, the non-parametric Kruskal–Wallis test followed by the Dunn posttest was used to compare all pairs of data.

InStat GraphPad Prism 6.0 software for Windows was used for the statistical analysis.

## Results

### Th17 Cells

**Figure 2:**
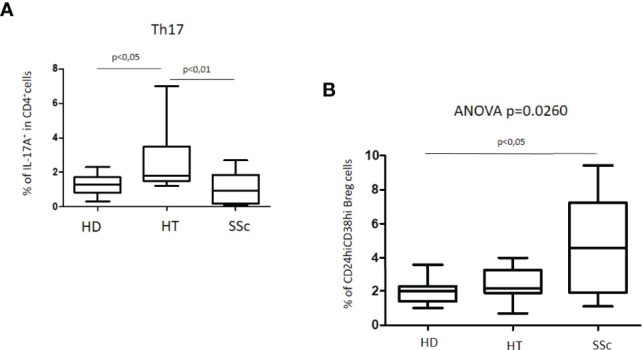
**(A)** Percentage of Th17 cells in patients with SSc as compared with both HT patients and healthy donors (HD). **(B)** Percentage of unstimulated CD24hiCD38hi Breg cells in patients with SSc as compared with both HT patients and healthy donors (HD). Kruskal–Wallis followed by Dunn posttest was used for multiple comparisons.

An increased percentage of Th17 cells was observed in HT patients as compared with both healthy donors (HD) and patients with SSc (Kruskal–Wallis test, p = 0.0018). The simultaneous presence of HT in patients with SSc did not modify these differences (not shown). No significant differences were observed instead between HD and SSc patients (**Figure 2A**).

### Unstimulated CD24^hi^CD38^hi^ B Cells

Total CD45^+^CD19^+^ B cells were similar in all groups of patients, also when compared with the HD group.

The characterization of CD24^hi^CD38^hi^ B regulatory cells, usually defined as transitional or immature phenotype, revealed peculiar differences. The percentage of this Breg cell phenotype in HT patients as compared to the HD group was similar. In the whole group of SSc patients, the percentage of CD24^hi^CD38^hi^ Breg cells was higher than in both HD (4.6% versus 2.0%) and HT (4.6% versus 2.2%) (ANOVA test: p = 0.0260) (**Figure 2B**). A difference was also observed in isolated SSc patients as compared with HD (p = 0.0330) (not shown). No significant differences were observed instead among patients with isolated SSc and those with SSc+HT.

### CpG Oligonucleotide-Stimulated CD24^hi^CD38^hi^ B Cells

**Figure 3:**
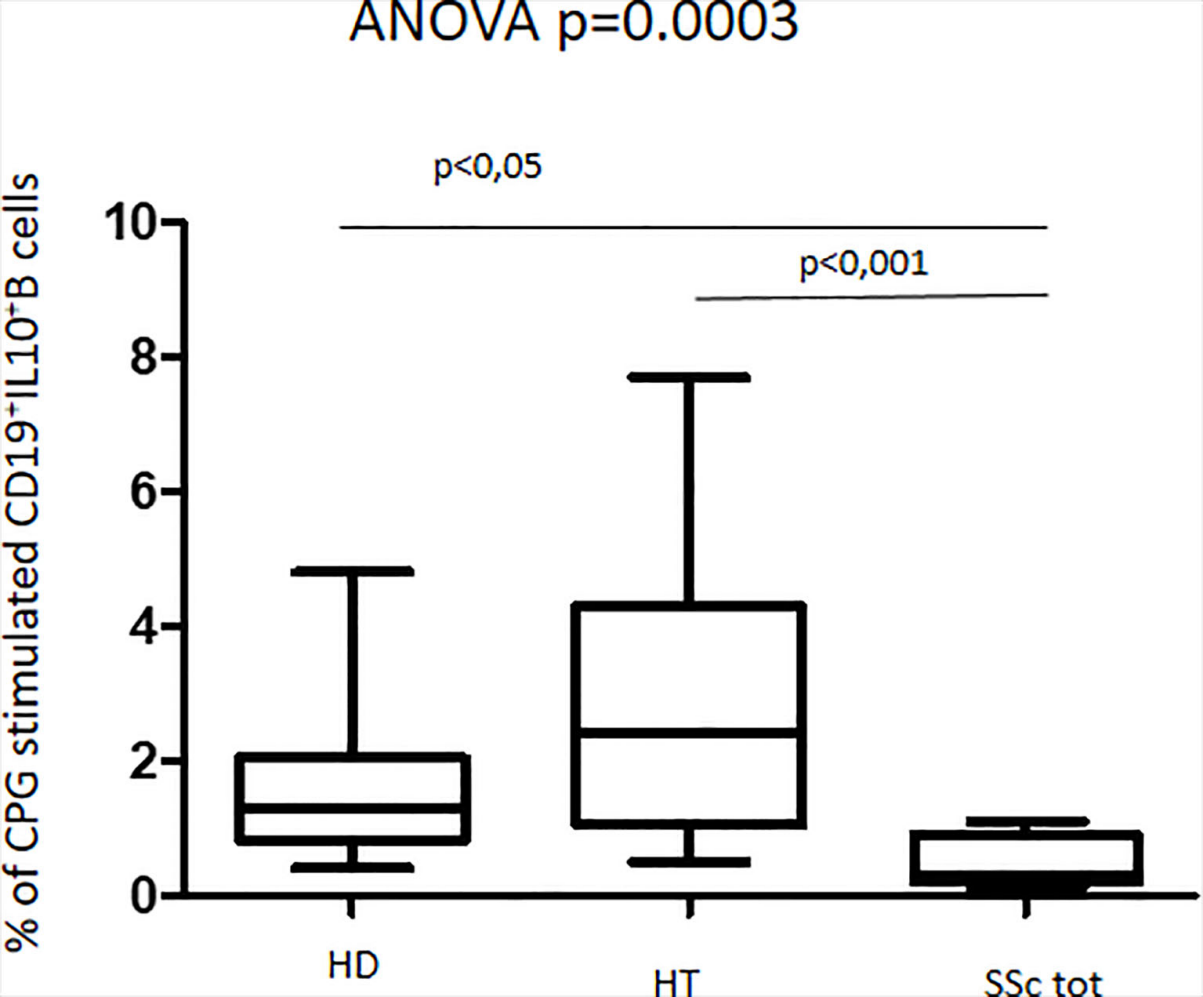
Percentage of CpG-stimulated CD19^+^IL10^+^B cells in patients with SSc as compared with both HT patients and healthy donors (HD). Kruskal–Wallis followed by Dunn posttest was used for multiple comparisons.

Upon culturing PBMCs in the presence of CpG oligonucleotides, we observed similar CD19^+^ cell proportions among all groups, as detected in basal conditions (data not shown). Levels of IL-10-producing B cells (CD45^+^CD19^+^IL10^+^) were significantly increased in HT patients as compared to both HD and total SSc patients (ANOVA test, p = 0.0003) (**Figure 3**). Analyzing SSc patient samples, the cell percentages appeared to be similar in both isolated SSc and HT+SSc patients (0.5% vs. 0.2%; p = 0.6187) (not shown). Similarly, no differences were observed between HT patients and healthy donors (2.4% vs. 1.3%; p = 0.1720).

**Figure 4:**
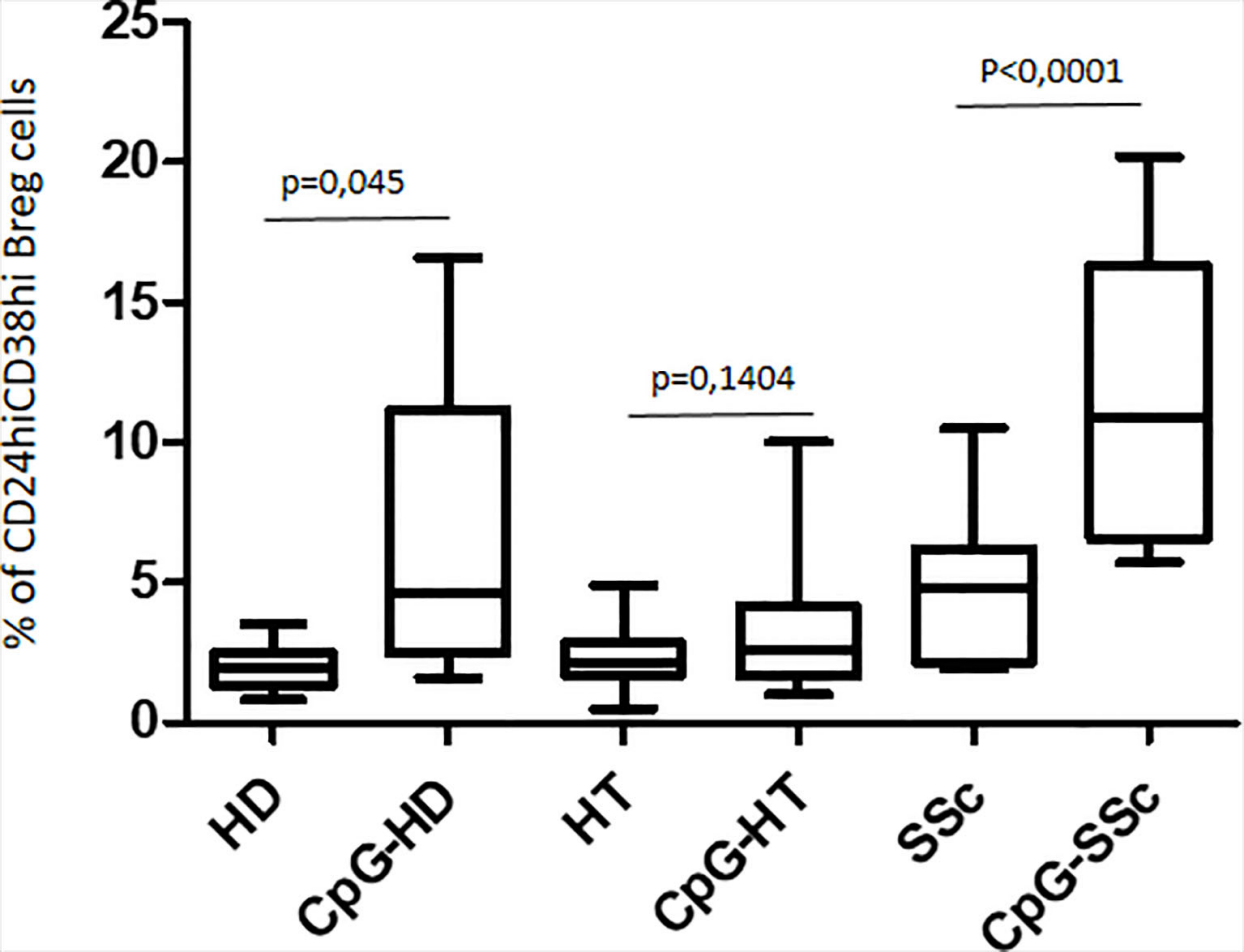
Comparison of the effects of CpG stimulation on CD24hiCD38hi Breg cells in healthy donors (HD), in HT patients, and in patients with SSc. Paired T test has been used for single comparisons.

**Figure 5:**
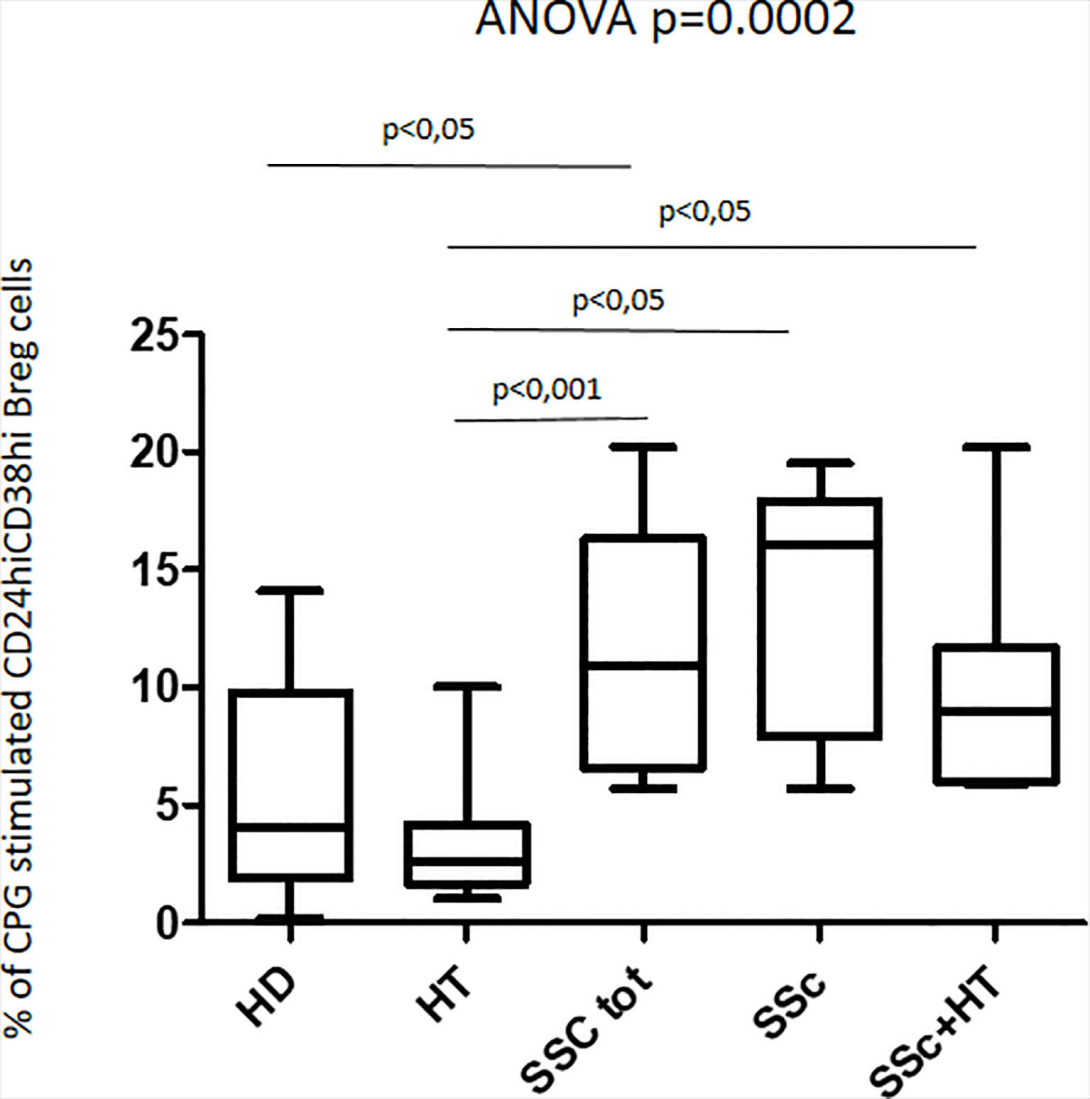
Percentage of CpG-stimulated CD24hiCD38hi Breg cells in the whole patient sample with SSc, in those with isolated SSc, and in those with SSc+HT as compared with both patients with isolated HT and healthy donors (HD). Kruskal–Wallis followed by Dunn posttest was used for multiple comparisons.

The response to CpG oligonucleotide stimulation was characterized by a different increase in CD24^hi^CD38^hi^ B-cell percentage in the different groups. In particular, the median increase was higher in patients with SSc (10.9% vs. 4.9%; p < 0.0001) than in the HD group (3.7% vs. 2.0%; p = 0.045) and in HT patients (2.6% vs. 2.2%; p = ns) (**Figure 4**). When the effect of CpG stimulation was analyzed in the different groups, a brisk increase in CD24^hi^CD38^hi^ B-cell percentage was observed in patients with SSc as compared to HD, irrespective of the concurrent presence of Hashimoto’s thyroiditis (**Figure 5**). This finding is in keeping with the evidence that, in patients with isolated HT, the percentage of this B phenotype was similar to that of healthy donors (**Figure 5**).

**Figure 6:**
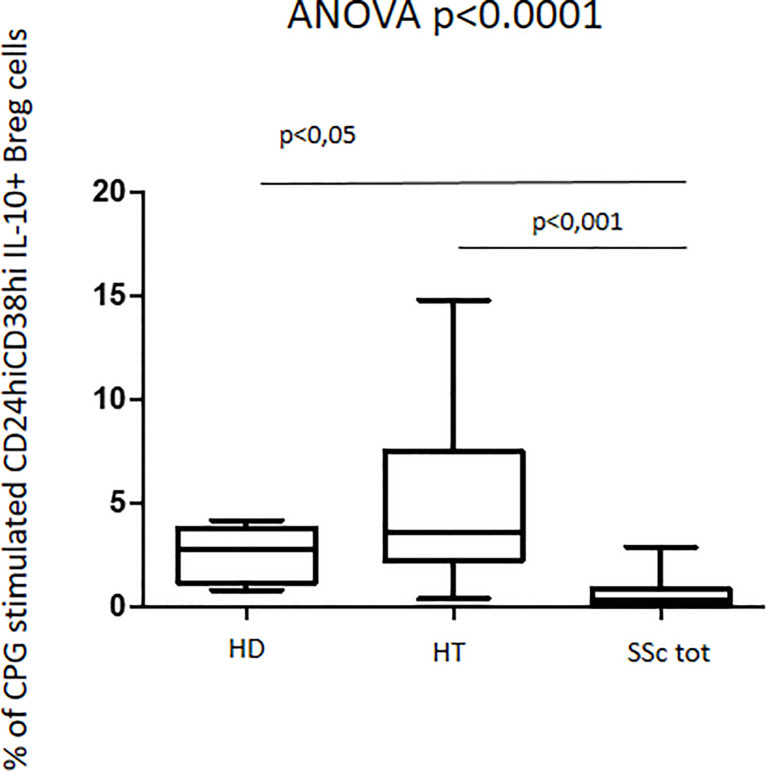
Percentage of CpG-stimulated CD24hiCD38hi IL10^+^ Breg cells in patients with SSc as compared with both HT patients and healthy donors (HD). Kruskal–Wallis followed by Dunn posttest was used for multiple comparisons.

In contrast with these quantitative findings, upon CpG stimulation, qualitative data analysis of the fraction of regulatory B cells producing IL-10^+^ showed entirely opposite results. In fact, HT patients showed a slightly increased percentage of CD24hiCD38hi IL10^+^ B regulatory cells as compared to the one observed in healthy donors (4.1% vs. 2.8%). Interestingly, this phenotype appeared significantly decreased in patients with SSc when compared to both HT patients and healthy donors (ANOVA: p < 0.0001) (**Figure 6**). This decrease was observed in both isolated SSc and HT+SSc showing no difference between these two groups of patients (p = ns) (not shown).

## Discussion

Systemic sclerosis (SSc) is a systemic autoimmune disease characterized by an extensive connective tissue involvement (1) along with a B-lymphocyte activation (14). Hashimoto’s thyroiditis (HT) often occurs associated with SSc (7–9, 11, 12); several studies have focused on the immunological characteristics of isolated SSc or HT (13, 15, 18, 20–23, 28–30), but it is unknown whether these two autoimmune disorders share some of the immunological mechanisms.

As expected, our data showed that in the HT group the percentage of Th17 lymphocytes was significantly higher than in healthy donors (13, 16, 20, 25). However, the percentage of Th17 cells in HT patients was significantly higher as compared to that in SSc patients. In the latter, the Th17 pathway directly correlates with the inflammatory state as measured by the increase in autoantibody production (15, 23). In our study, the SSc patients were enrolled based on the remission state of the disease, as supported by the absence of any immune-modulatory treatment, and this may explain the absence of a significant increase in Th17 cells as related to the decrease in their state of activity.

In the present study, before stimulation, CD24^hi^CD38^hi^ Breg cells were higher in the SSc patients than in the HD and HT groups; these data are in keeping with those previously described in patients with organ-specific autoimmune disorders associated with Hashimoto’s thyroiditis (20). However, other studies have shown that the percentages of CD24^hi^CD38^hi^ and CD24hiCD27+ B cells were significantly reduced in SSc as compared with those in healthy controls (15, 23). In these studies, the lack of Breg population was directly related with an increase in proinflammatory circulating T cells and with a worse clinical prognosis of SSc. Again, the remission stage of the disease of our patients with SSc may have prevented the rise in Th17 cells.

One of the main signs of Breg activation is the detection of their IL10 production which characterizes the Breg effector functions. In fact, this marker has been studied in naïve cells and upon an appropriate stimulus (20–22). In a previous study, it has been demonstrated that CpG oligonucleotide stimulation determines the increase in IL10^+^CD24^hi^CD38^hi^ Breg cells in both HT and HD subjects (20). In the present study, following stimulation with CpG, Breg cells were almost triplicate in patients with SSc (with or without HT) as compared with healthy controls and isolated HT. However, despite this higher percentage of Breg, the percentage of cells producing IL-10 was significantly reduced in patients with SSc (with or without HT) when compared to those in healthy subjects and in isolated HT. This finding suggests that in patients with systemic sclerosis, the number of naïve B and IL10^+^Breg phenotypes is increased independently from the coexistence of HT, despite their reduced functionality (12, 15, 23). The prevailing effect of autoimmune-associated diseases over the one of HT has been already described for celiac disease but in an opposite fashion (21). The lack of Breg functions in SSc patients has been described in the active phase of other rheumatic diseases (31–33) or in progressive SSc (15, 23, 34, 35). A similar situation has also been described in systemic lupus in which the lack of IL-10 production by immature Breg cells (33) and an inverse correlation between mature Breg cells and disease activity have been observed (31).Whether this reduction of functional Breg also determines an impairment of their immunosuppressive function is not completely known. In light of the quiescent phase of the SSc in our study group, we may speculate that CD24^hi^CD38^hi^ may exert their suppressive role by using a different pathway than IL10, such as IL35 or TGF-β (36–38). The existence of variable functional expressions of the different Breg populations on the basis of the state of disease activity may thus be postulated (15, 23, 31, 34) and could have therapeutic implications. Indeed, studies conducted on humans and animals have shown that therapy with anti-CD20 autoantibody (rituximab), which determines a total B-cell depletion, could have different effects depending on the activity state of the disease (18, 19). In a mouse model of systemic autoimmunity, it has been shown that depletion of B lymphocytes in the terminal phase of the disease could be effective, while in the initial phase it could exacerbate clinical manifestations (39, 40). This evidence may suggest a different composition of B-cell populations in the course of the disease. It is possible that at the beginning of the disease, the Breg population is prevalent as compared to the effector B one, while with the progression of the disease the chronic inflammatory state could deviate the differentiation of B cells toward the B effector (41, 42).

Breg cells suppress immunopathologic phenomena, by avoiding the expansion of pathogenic T cells and pro-inflammatory processes, through the production of interleukin-10, IL-35, and transforming growth factor β (41). Since SSc involves the upper digestive tract and at that level most of immune signaling takes place, it is obvious to look at an interaction between the behavior of Breg cells, the resident microbiota composition, and the autoimmune disorders. While the linkage between autoimmune thyroid disorders and gut microbiota (43, 44) and Breg cells is well known (20–22), data on the SSc–gut microbiota interplay are scarce. Enteric bacteria activate IL-10^+^ intestinal B cells that are able to maintain mucosal homeostasis in an *in vitro* mouse model (45). Despite that, most of evidence in humans supports the role of regulatory B cells in autoinflammatory disorders, preventing bacterial antigen-driven colitis in humans and in mouse models (see for rev. 46).

*In vivo*, a greater percentage of Lactobacillus and Streptococcus and a reduced presence of Sutterella in the microbiota composition of SSc patients as compared to HCs have been described (46). Furthermore, the possibility of a dysbiotic state in our SSc patients might be supposed based on the increased need for levothyroxine that characterized these patients. In fact, a role for intestinal bacteria in the enterohepatic recycling of thyroid hormones has been postulated (47), thus affecting the pharmacologic homeostasis of levothyroxine (24).

The strength of our study is related to the peculiar sample of poly-autoimmune patients bearing a systemic disorder associated with Hashimoto’s thyroiditis. The quiescent phase of SSc in our patients may highlight the modified behavior of Breg cells in the different stage of diseases.

Besides the small number of our samples, related to the rarity of these autoimmune associations, the lack of a possible comparison with SSc patients during the active phase of the disease represents the main limitation of this study.

In summary, these findings indicate that the presence of SSc, either isolated or associated with HT, seems to be characterized by an enrichment of total Breg cells but a reduced Breg IL-10-producing phenotype, representing functional Breg. This last finding was entirely due to the presence of SSc since the co-presence of HT did not change the results. This behavior is different from the ones described about the association of HT with organ-specific autoimmune disorders.

## Data Availability Statement

The raw data supporting the conclusions of this article will be made available by the authors, without undue reservation.

## Ethics Statement

The studies involving human participants were reviewed and approved by Local Ethical Committee (ASL C, Roma- Latina, Italy). The patients/participants provided their written informed consent to participate in this study.

## Author Contributions

MC, GR, and CV designed the study and contributed to the manuscript. SF, AA, and PF screened and selected the patients with SC and contributed to the manuscript. GM and IG performed the analysis and validated the results. SC and MS performed statistical analysis, wrote the manuscript, and revised the pertinent literature. All authors revised and approved the final manuscript.

## Funding

This study has been supported by Sapienza University of Rome, Italy (Progetti di Ateneo 2017 and 2018 n.RM11715C71A02A85).

## Conflict of Interest

The authors declare that the research was conducted in the absence of any commercial or financial relationships that could be construed as a potential conflict of interest.

## Publisher’s Note

All claims expressed in this article are solely those of the authors and do not necessarily represent those of their affiliated organizations, or those of the publisher, the editors and the reviewers. Any product that may be evaluated in this article, or claim that may be made by its manufacturer, is not guaranteed or endorsed by the publisher.
